# Posterior Vaginoplasty With Perineoplasty: A Canadian Experience With Vaginal Tightening Surgery

**DOI:** 10.1093/asjof/ojz030

**Published:** 2019-10-15

**Authors:** Ryan E Austin, Frank Lista, Peter-George Vastis, Jamil Ahmad

**Affiliations:** 1 Division of Plastic and Reconstructive Surgery, Department of Surgery, University of Toronto,, Toronto, Ontario, Canada; 2 Medical School, Royal College of Surgeons in Ireland, Dublin, Ireland

## Abstract

Following vaginal trauma, most commonly vaginal delivery, women may experience vaginal laxity as a result of local tissue stretching and separation of the pelvic floor musculature. In addition to this generalized sensation of laxity, women may complain of decreased sexual satisfaction, gaping of the perineum, and excessive vaginal secretions. Since 2014, the authors have used a posterior vaginoplasty with perineoplasty technique for the surgical management of vaginal laxity. To date, the authors have performed surgical vaginal tightening in 30 consecutive patients and found that the posterior vaginoplasty with perineoplasty technique has allowed us to achieve reproducible outcomes with no postoperative complications. This article will review the authors’ approach to patients presenting for surgical vaginal tightening and the authors’ experience to date, including our preoperative screening, perioperative management, and detailed steps of the procedure.

Female genital cosmetic surgery (FGCS) is one of the fastest growing areas in aesthetic plastic surgery.^1^ When performed in the appropriate patients, FGCS procedures have high satisfaction and low complication rates.^2–5^ Although many plastic surgeons offer FGCS treatment options for the external female genitalia, few provide vaginoplasty and perineoplasty.

Following vaginal trauma, most commonly vaginal delivery, women may experience vaginal laxity due to local tissue stretching and separation of the pelvic floor musculature. Women may complain of altered sexual satisfaction, decreased friction during intercourse, altered vaginal sensation, and a generalized feeling of laxity.^4,6,7^ Furthermore, women commonly complain of gaping of the vaginal vestibule, which creates an aesthetic deformity allowing visibility of the vaginal mucosa.^8^ This gaping also creates several functional concerns including increased/excessive vaginal secretions due to mucosa exposure, altered ability to achieve orgasm, and vaginal air entrapment resulting in embarrassing sounds during sexual intercourse.^9^

As public awareness regarding treatment options for vaginal laxity has increased, vaginal tightening procedures have increased in popularity. Although these procedures have been associated with high patient and partner satisfaction,^4-6,10-13^ descriptions of vaginoplasty techniques for this indication are limited as are data regarding patient safety and outcomes.^2,11,13-16^

Since 2014 we have exclusively used a posterior vaginoplasty with perineoplasty technique to address concerns related to vaginal laxity. This article reviews our approach to patients presenting for vaginal tightening and our experience with the procedure to date.

## PREOPERATIVE CONSIDERATIONS

Vaginal laxity is a sensitive and highly personal issue. Consultation should take place in a private, comfortable setting and should not be rushed. To ensure patient comfort, we prefer all discussion take place with the patient fully clothed. Patients change into a gown only for physical examination; they are invited to re-dress prior to further discussion taking place.

A complete history should be obtained from the patient including past medical and surgical history (including any prior nonsurgical treatments for vaginal tightening), medications, allergies, and smoking status. A focused gynecological history must also be obtained including: obstetrical history and method of delivery, menstrual history, current contraceptive use, history of urinary incontinence, history of abnormal cervical cytology (including date of last Pap test), history of hemorrhoids, and any history of prior vaginal trauma, sexual dysfunction, sexually transmitted infection, pelvic malignancy, or pelvic pain disorder.

It is also important to develop an understanding of the patients’ concerns and motivations for seeking vaginal tightening surgery. We specifically review these common complaints with patients preoperatively and document any additional concerns that are reported. In some instances, a patient’s primary motivation for surgery is decreased sexual satisfaction of their partner. In these cases, it is important to remind the patient there is no guarantee that vaginoplasty will affect their partners sexual satisfaction. The decision to undergo vaginoplasty must always be a personal choice made of the patients own volition. If concern exists that a patient is being pressured into surgery, is using surgery to remedy relationship issues, or if a patient has unrealistic expectations, this must be identified and addressed during the preoperative consultation.

A physical examination of the external genitalia should be performed, including a bimanual pelvic examination. We advise all surgeons to bring a chaperone into the room for the duration of the physical examination, both for patient comfort and for medicolegal protection. The degree of vaginal laxity, the presence of posterior gaping or webbing of the vaginal vestibule, perineal body length (ie, distance between the anus and posterior fourchette), and the presence of hemorrhoids are documented. It is important to assess for any pelvic masses or pelvic organ prolapse. Patients with a history of untreated pelvic organ prolapse, cystocele, or rectocele should be referred to a gynecologist for further evaluation.^14^ If concern exists regarding the degree of scarring between the posterior vagina and anterior rectum, digital rectal examination may be performed with patient consent. If a gliding tissue plane exists (ie, the ability independently mobilize the two surfaces over one another), posterior vaginoplasty is a suitable technique.

Contraindications to posterior vaginoplasty with perineoplasty include active or untreated pelvic infection or inflammatory process, current pregnancy, pelvic malignancy, bleeding disorders, and unrealistic expectations of surgical results. History of vulvodynia, dyspareunia, or chronic pelvic pain are considered relative contraindications to surgery.^17^

Potential surgical risks of vaginoplasty are reviewed with all patients preoperatively, including bleeding, hematoma, infection, wound dehiscence, urinary retention, injury to bowel/bladder, development of a rectovaginal fistula, scarring, vaginal stenosis, dyspareunia, altered sensation, and an inadequate surgical result.^3,4,14^

## SURGICAL TECHNIQUE

Vaginoplasty with perineoplasty is performed as a day surgery procedure under general anesthesia. Following administration of prophylactic antibiotics, the patient is placed in the lithotomy position in stirrups. Care is taken during positioning to pad all pressure points to prevent iatrogenic injury. All patients wear compression stockings during the perioperative period and intermittent pneumatic compression devices are placed intraoperatively to provide mechanical VTE prophylaxis. Preoperative photographs are taken for documentation at this time. The vaginal canal, perineum, and proximal thighs are prepared with 10% povidone-iodine solution and draped in a sterile fashion.

**Figure 1.**
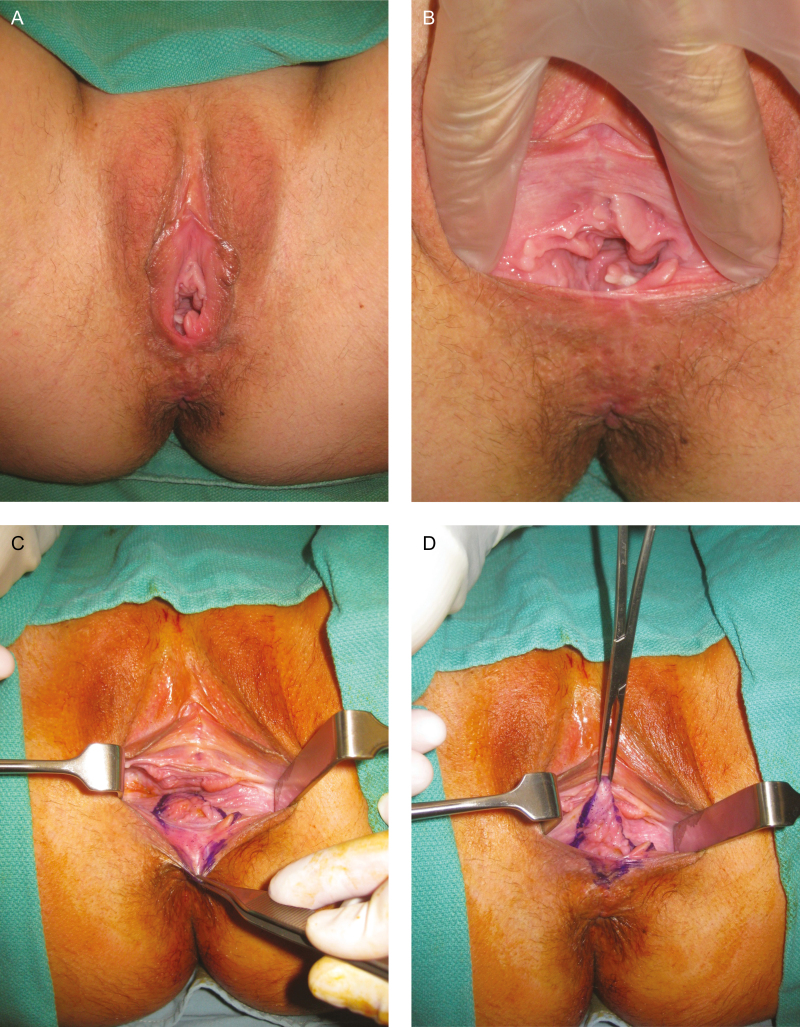

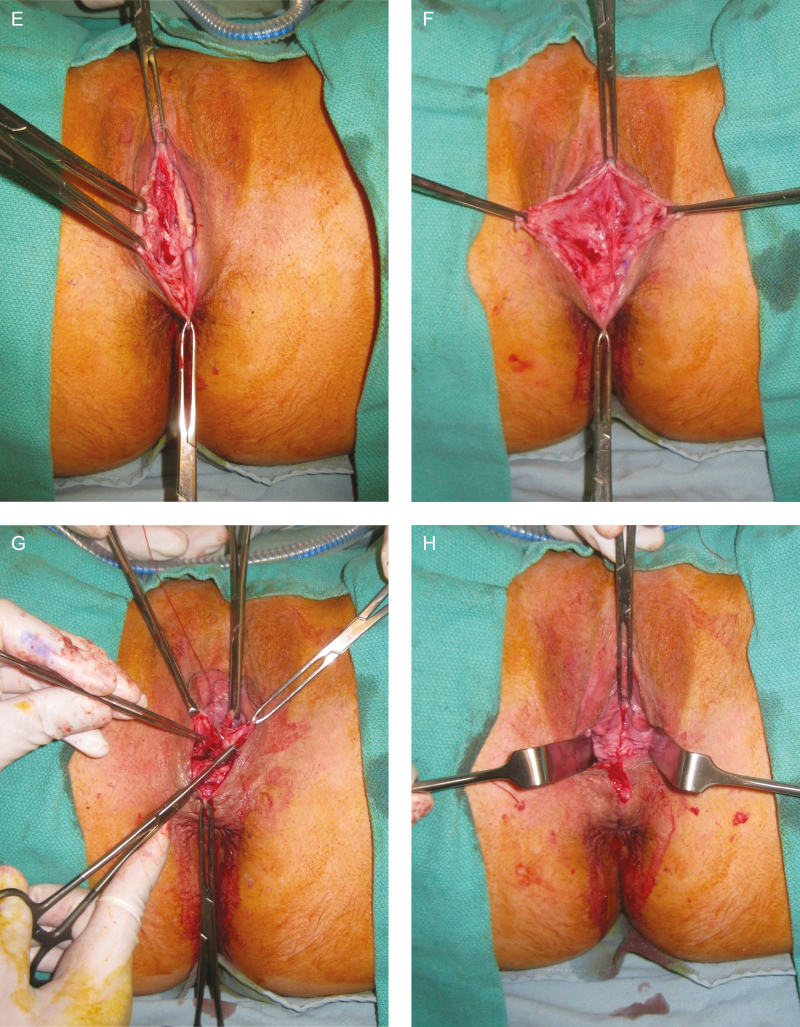

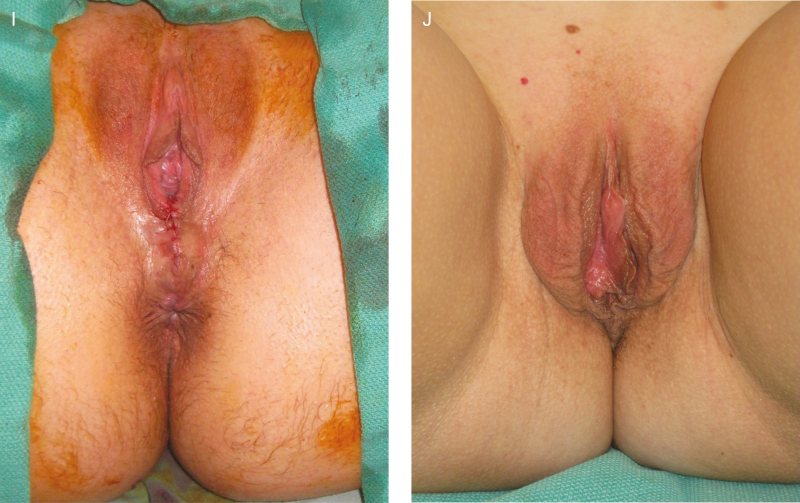
(A) Preoperative view of a 34-year-old woman presenting with complaints of vaginal laxity. She had two previous vaginal deliveries. The common complaints of patients presenting for vaginal tightening are visible including increased visibility of the vaginal mucosa, irregularity of the vaginal mucosa, and separation of the posterior fourchette with shortening of the perineal length. This woman also complained of moderate stress urinary incontinence. (B) Retraction of the introitus demonstrates increased soft tissue laxity of the vaginal vestibule. (C) Markings for posterior vaginoplasty with perineoplasty are designed as two mirror-image triangular-shaped excisions. (D) An Allis clamp placed at the superior tip of the internal triangle resection pattern (4–6 cm inferior to the posterior fornix) combined with long right-angled retractors provides visualization and access to the posterior vaginal wall. (E) Dissection of the posterior vaginal wall. Note the scar tissue present in the midline, near the unelevated (left) posterior wall. The submucosal plane laterally can be easier to develop with careful finger-sweep using a gauze. (F) Once both mucosal flaps have been elevated to the level of the planned resection, they can be amputated to allow for direct reapproximation and repair. (G) During the posterior vaginal wall repair, submucosal sutures are oriented parallel to the mucosal surface to avoid injury to the rectum and the perirectal venous plexus. (H) After reapproximation of the bulbospongiosus and superficial transverse perineal muscles, the diameter of the introitus and vaginal canal is significantly narrowed. (I) Immediate postoperative appearance. (J) Nine-month follow-up photograph demonstrates correction of preoperative perineal gaping with reconstitution of the posterior fourchette and increased perineal length. This patient also reported resolution of her stress urinary incontinence.

**Figure 2.**
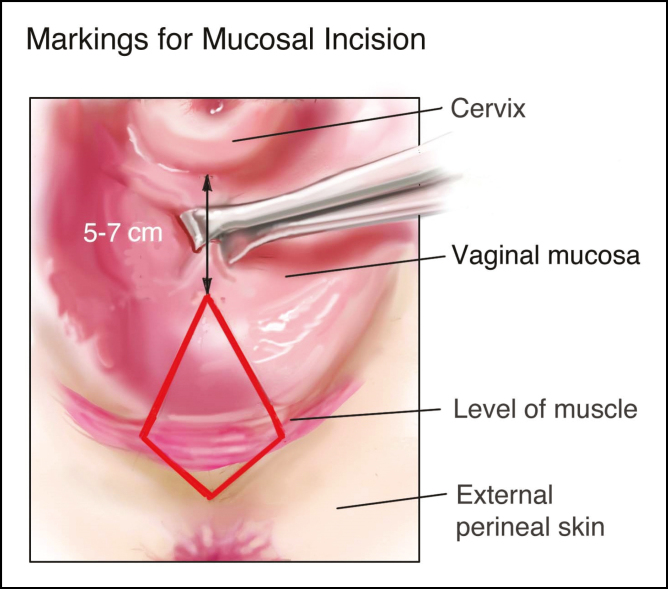
Illustration showing the surgical markings for the mucosal incision during posterior vaginoplasty.

The surgical markings for posterior vaginoplasty with perineoplasty are designed as two triangular-shaped resection patterns, one internal triangle along the posterior wall of the vaginal canal and one external triangle of the perineum (Figure 1A-D and Video 1, available as Supplementary Material online at www.asjopenforum.com). These two triangles meet at the level of the hymenal ring, corresponding to the widest point of planned excision (Figure 2). This gives the overall excision pattern a diamond-shape. The tip of the internal triangle is placed 4–6 cm inferior to the posterior fornix of the vagina. Hymenal remnants are usually identifiable and the medial edge of the two most midline remnants serve as a guide for the width of the planned resection at the level of the hymenal ring. The width of the resection can be confirmed by approximating the planned margins with tissue forceps to ensure they can be approximated with minimal tension. If there is any doubt as to the width of the planned resection, it is better to err on the side of under-resection to avoid overtightening and the risk of vaginal stenosis or dyspareunia. The external marking should transition from the perineal skin to the mucosa of the vaginal vestibule at a right angle. This transition point must be carefully selected at it will define the new position of the posterior fourchette.

Once the markings have been confirmed, an Allis clamp is placed at the tip of the internal triangle marking (Figure 1E). Combined with long right-angled Langenbeck retractors, this clamp will provide visualization and access to the posterior vaginal wall. Approximately 5–10 mL of 0.25% bupivacaine with epinephrine (1:400,000) is injected in the submucosal plane throughout the planned resection areas. Starting at the hymenal ring, a short, superficial midline incision is made in the posterior vaginal mucosa using a 15-blade scalpel. Blunt scissor dissection is used to develop a submucosal plane in the midline, and the posterior vaginal mucosa is split along the midline to the tip of the internal triangular marking. Care must be taken to stay in the submucosal plane during midline dissection, as deeper dissection risks injury to the vast perirectal venous plexus and the anterior rectum itself. Scar tissue from prior trauma is most dense in the midline and can make midline dissection more difficult. If concern about the amount of tissue between the vagina and rectum, intraoperative digital rectal examination can be performed to confirm tissue thickness and a gliding plane between these surfaces.

Once the midline mucosa has been split, mucosal flaps are raised laterally in the submucosal plane. Flaps are raised to the level of the planned mucosal resection (Figure 1F). Midline dissection is performed using blunt and sharp scissor dissection, but once dissection proceeds lateral to the scar tissue the submucosal plane is more easily defined with careful finger dissection using a gauze to sweep the mucosa off the underlying tissue. Placement of Allis clamps along the medial edge of the mucosal flap aid in retraction and dissection. The redundant mucosa is sharply resected using scissors.

**Figure 3.**
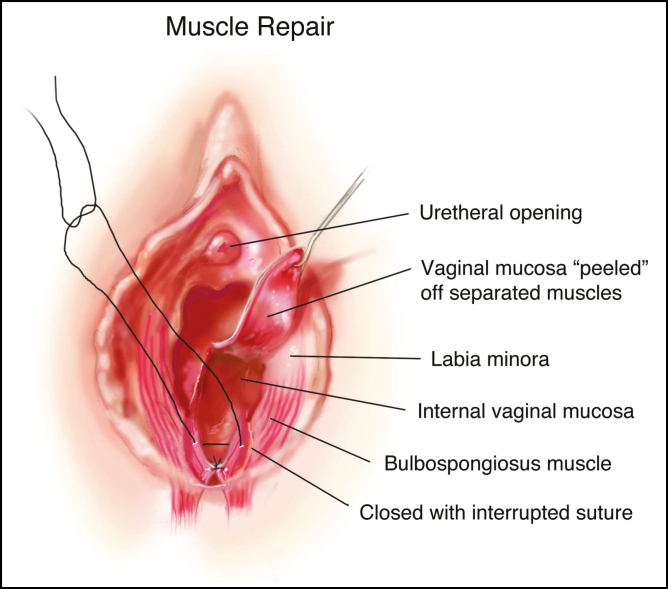
Illustration demonstrating the muscle repair and related anatomy.

Proximal to the level of the hymenal ring, the perineal skin and vaginal mucosa is sharply resected along with underlying scar tissue (Figure 3). This will expose the underlying pelvic musculature separated as a result of prior vaginal delivery, namely, the bulbospongiosus muscle at the hymenal ring and the superficial transverse perineal muscle in the perineum.

**Figure 4.**
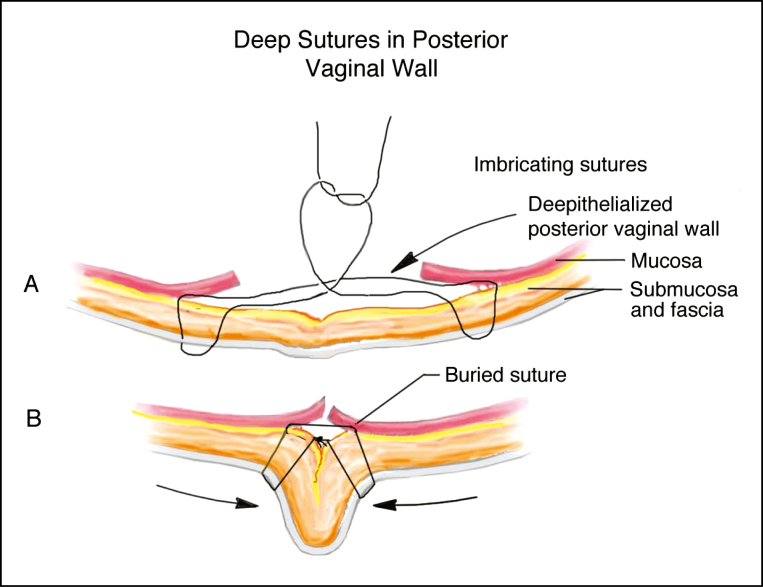
(A, B) Illustration demonstrating the placement of deep sutures in the posterior vaginal wall.

Once hemostasis has been ensured, repair of the posterior vaginal wall proceeds from internal to external (Figure 4). The submucosal layer at the apex of the resection is reapproximated with a deep layer of interrupted 2-0 Vicryl sutures (Johnson & Johnson, Markham, ON, Canada). These sutures should be oriented parallel to mucosal surface (Figure 1G). This suture orientation not only avoids deep suture bites to minimize the risk of accidental injury to the rectum or the perirectal venous plexus, but also allows the suture to grasp the fascia of the underlying pelvic floor musculature, thus providing strength to the deep repair. This deep repair continues in this manner to the level of the hymenal ring. The overlying mucosa is then reapproximated with a layer of interrupted or running 3-0 Vicryl Rapide sutures (Johnson & Johnson, Markham, ON, Canada) (Figure 1H).

Starting at the level of the hymenal ring and into the perineum, deep suture orientation transitions to traditional inverted deep sutures; with additional muscle bulk in this region the distance between the vagina, the rectum and the anal canal is significantly greater. Once the scar and excess tissue have been removed at the hymenal ring, the separated edges of the bulbospongiosus muscle are identified and reapproximated using multiple deep interrupted 2-0 Vicryl sutures. This should create a vaginal canal diameter of 2.5–3.5 cm, which will accommodate digital examination with approximately two fingers. Overtightening should be avoided as this may result in vaginal stenosis and permanent dyspareunia. In the perineum, repair of the superficial transverse perineal muscles is performed in a similar fashion. Perineal repair will reconstruct and lengthen the perineal body, reconstitute the posterior fourchette, and correct posterior gaping of the vaginal vestibule. Again, care must be taken to prevent overtightening to minimize the risk of dyspareunia. Once the muscle repairs have been completed, skin and mucosa are repaired with interrupted 3-0 Vicryl Rapide sutures (Figure 1I-J).

At the completion of the procedure, the vaginal canal is packed with a petroleum jelly coated 1″ ribbon gauze. No additional dressing is required, though patients are instructed to wear a pad to prevent blood staining of their clothing. All patients are provided with a prescription for multimodal analgesia, including acetaminophen (1000 mg PO q8h × 5 days), celecoxib (200 mg PO daily × 5 days), pregabalin (25 mg PO q12h × 5 days), and hydromorphone (1–2 mg PO q6h prn). Patients are also provided with a postoperative prescription for senna glycoside (1–2 tablets PO qhs prn) to prevent constipation. We do not routinely prescribe antibiotics postoperatively. All patients return for follow-up on postoperative day one and the packing is removed. Patients are instructed to gently rinse the perineum with soapy water four times daily and after every washroom visit to keep the area clean, minimize the risk of infection, and prevent scabs from forming along the incision. Patients return for follow-up appointments at the 1-week, 2-week, 1-month, and 3-month time points. If any external perineoplasty sutures remain at the 2-week visit, these are removed. Patients are instructed to refrain from strenuous physical activity for 4 weeks and to avoid tampon use and sexual intercourse for 6 weeks. Vaginal dilators are not used postoperatively.

## EXPERIENCE AND OUTCOMES

**Figure 5.**
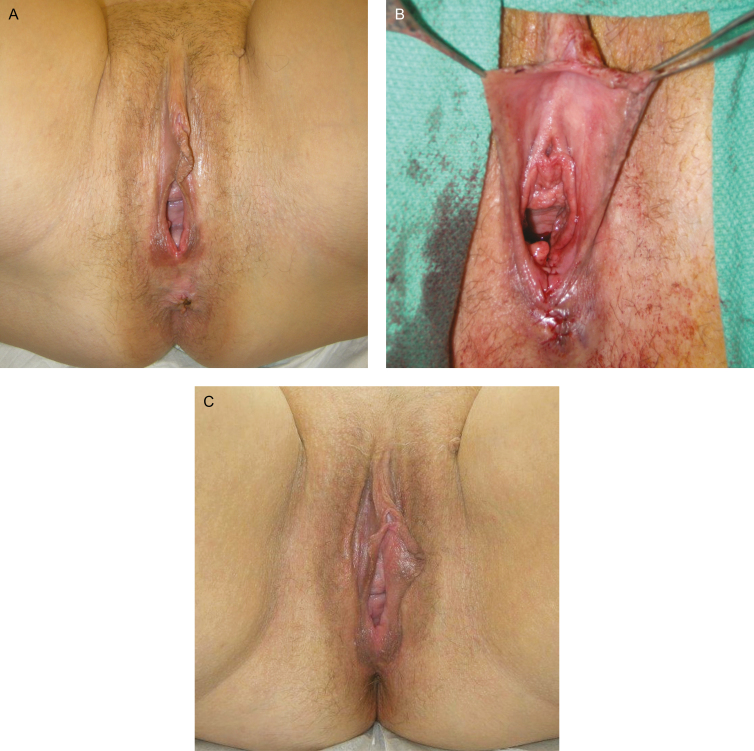
(A) Preoperative view of a 52-year-old woman presented with concerns of vaginal laxity. She had two previous vaginal deliveries. Additionally, she complained of stress urinary incontinence. (B) Immediate and (C) 6-month postoperative views after posterior vaginoplasty and perineoplasty. Preoperatively, her Vaginal Laxity Questionnaire score was 1 (very loose) and at 3 months postoperatively, it was 4 (neither loose nor tight). Preoperatively, her International Consultation on Incontinence Questionnaire-Urinary Incontinence Short Form (ICIQ-UI SF) score was 11 and at 3 months postoperatively, it was 0.

A retrospective chart review was performed for all patients that underwent posterior vaginoplasty with perineoplasty at an aesthetic surgery practice. All patients presented with the primary complaint of vaginal laxity (Figure 5). The guiding principles of the Declaration of Helsinki were strictly applied and adhered to in this study.

A total of 30 patients underwent vaginal tightening using this technique between June 2014 and February 2019. Average patient age was 41 years (range, 23–58 years) with an average body mass index of 23.7 kg/m^2^ (range, 18.8–29.8 kg/m^2^). The majority of patients (80.0%) were nonsmokers. Amongst the cohort, the majority (86.7%) of patients were multiparous, with the remainder (13.3%) being primiparous. Only one patient (3.3%) never had a spontaneous vaginal delivery. Average operative time for posterior vaginoplasty with perineoplasty was 68 min (range, 40–92 min). Fourteen patients (46.6%) underwent a total of 17 additional procedures at the time of their vaginoplasty procedure (Table 1). Average length of follow-up was 233 days (range, 19–1,311 days). There were no complications noted in any case. Two patients (6.7%) did state that despite experiencing an improvement of their preoperative symptoms, they would have liked to feel tighter. One of these patients underwent a revision procedure under local anesthetic to achieve further tightening.

**Table 1.** List of Aesthetic Surgical Procedures Performed at the Same Time as Posterior Vaginoplasty With Perineoplasty

Procedure	No. of procedures
Labiaplasty	8
Mastopexy-augmentation	4
Breast augmentation	2
Rhinoplasty	1
Abdominoplasty	1
Mastopexy	1
Abdominal scar revision	1

In an effort to better quantify outcomes in the treatment of vaginal laxity, all patients undergoing surgical and nonsurgical vaginal tightening now complete patient-reported outcome measures, specifically the Female Sexual Function Index (FSFI)^18^ and the International Consultation on Incontinence Questionnaire—Urinary Incontinence Short Form (ICIQ-UI SF),^19^ prior to treatment as well as at the 3- and 6-month postoperative time points. Data from these self-reported outcome measures are limited at this time, given that these questionnaires were not completed by all patients in the study cohort.

## CONCLUSION

This article presents a reliable and reproducible approach to the surgical management of vaginal laxity. Based on our experience, posterior vaginoplasty with perineoplasty has demonstrated high patient satisfaction with no complications. Interestingly, almost half of the patients in our review underwent additional aesthetic surgical procedures at the time of their vaginal tightening procedure. This represents an area of tremendous potential growth for plastic surgeons. As patient knowledge of treatment options for vaginal laxity increases, demand for vaginoplasty will continue to grow. Posterior vaginoplasty with perineoplasty would be a complementary procedure for any plastic surgeon performing FGCS procedures.
